# Complete Genome Sequence of Burkholderia gladioli Phage Maja

**DOI:** 10.1128/MRA.01430-20

**Published:** 2021-02-04

**Authors:** Zihao Yu, Guichun Yao, Maria Guadalupe Vizoso-Pinto, Lichang Sun, Ry Young, Carlos Gonzalez, Mei Liu

**Affiliations:** a Center for Phage Technology, Texas A&M University, College Station, Texas, USA; b Infection Biology Lab, Instituto Superior de Investigaciones Biológicas, National Council of Scientific and Technological Research, National University of Tucuman, San Miguel de Tucumán, Argentina; c Institute of Food Quality and Safety, Jiangsu Academy of Agricultural Sciences, Nanjing, People’s Republic of China; DOE Joint Genome Institute

## Abstract

Burkholderia gladioli is a Gram-negative bacterium associated with cystic fibrosis infections. Here, we describe the genome sequence of *B. gladioli* phage Maja. Maja is most related to another *Burkholderia* phage, BcepF1, and may be a temperate phage, despite the absence of repressor or integrase homologs in its genome sequence.

## ANNOUNCEMENT

Burkholderia gladioli is a Gram-negative bacterium formerly classified as *Pseudomonas* (1). Although originally regarded essentially as a phytopathogen (2), *B. gladioli* can also colonize the lungs of cystic fibrosis patients (3, 4). Additionally, *B. gladioli* and the closely related species B. cepacia are associated with multidrug resistance (5). For the purpose of finding phage for therapeutic applications, *B. gladioli* phage Maja was isolated, and its genome annotation was performed.

Maja was isolated in 2019 from a soil sample from Hermann Park in Houston, TX (GPS coordinates, 29.7135373, −95.3910571). The filtered soil extract (soil sample mixed with phosphate-buffered saline [PBS] buffer) was enriched overnight against a *B. gladioli* clinical isolate (strain BgPK) in tryptic nutrient broth at 37°C. Phage was purified and propagated from the enrichment using *B. gladioli* strain BgPK as the host on tryptic nutrient broth agar at 37°C by the soft-agar overlay method (6). Phage genomic DNA was extracted from the precipitated phage particles and purified using a Wizard DNA cleanup kit as previously described (7). DNA libraries were prepared using an Illumina TruSeq Nano kit with 300-bp inserts. Genome sequencing was performed on an Illumina MiSeq instrument using v2 300-cycle chemistry. FastQC (www.bioinformatics.babraham.ac.uk/projects/fastqc) and FastX-Toolkit v0.0.14 (http://hannonlab.cshl.edu/fastx_toolkit/download.html) were used for the quality control and trimming of a total of 383,974 sequence reads. *De novo* genome assembly was done by SPAdes v3.5.0 (8). As a result, a single contig was assembled with 270.6-fold sequence coverage. The closure of the genome sequence was done by PCR (forward, 5′-CTTAGAAAGCCGCCCATAGA-3′; reverse, 5′-GGTATCGACATGGCGAAGAA-3′) and confirmed to be complete by Sanger sequencing. The structural annotation of the genome was performed with GLIMMER v3.0 (9) and MetaGeneAnnotator v1.0 (10). TransTermHP v2.09 was used for the prediction of rho-independent termination sites (11), and tRNAs were detected by ARAGORN v2.36 (12). Gene function prediction mainly relied on conserved domain searches with InterProScan v5.22 (13) and sequence similarity search by BLAST v2.2.31 at a 0.001 maximum expectation value (14). Specifically, the sequence similarity search was set at an E value of <0.001 against the NCBI nonredundant and Swiss-Prot/TrEMBL databases (15). Transmembrane domains were found and annotated using TMHMM v2.0 (16). Several structural predictions were performed with HHpred v3.2.0 (17). In addition, progressiveMauve v2.4 calculated genome-wide DNA sequence similarity to top BLAST nucleotide hits (18). All annotation tools (except HHpred) were used in the Center for Phage Technology Galaxy and Web Apollo platforms hosted at https://cpt.tamu.edu/galaxy-pub (19–21). All software was used with default parameters unless otherwise specified.

Phage Maja has a 68,393-bp genome sequence with 54.5% GC content. A total of 114 protein-coding genes were predicted, with a coding density of 92.4%. Overall, 33 protein coding genes were assigned a function. Comparative genomics and BLASTp analysis revealed that Maja is most related to another *Burkholderia* phage, BcepF1 (GenBank accession number NC_009015), both on the nucleotide level (56.8% nucleotide identity as determined by progressiveMauve) and on the protein level (sharing 90 similar proteins by BLASTp at E < 0.001), but Maja’s genome sequence contains a 15-kb region where the sequence identity is very low (not possible to align by BLASTn at E < 0.001) to all other known phages. Several structural genes were predicted, including the tail fiber, baseplate, and tail sheath proteins. In addition, two free-standing homing endonuclease genes, one GIY-YIG and the other HNH, were predicted in the genome sequence. Four lysis genes were identified, including two spanin genes, two endolysin genes, one glycosyl hydrolase, and one transglycosylase. Finally, many Maja genes exhibit high similarity to bacterial genes, suggesting that Maja may be temperate despite the absence of repressor or integrase homologs, or recently derived from a temperate ancestor, which could explain its low GC content (54.5%) compared to that of its host (∼68%) (22).

### Data availability.

The genome sequence of Maja is available in GenBank under accession number MT708549. The associated BioProject, SRA, and BioSample accession numbers are PRJNA222858, SRR11558342, and SAMN14609647, respectively.
